# Supplementary figures and images for: Diagnostic utility of quantitative analysis of microRNA in bile samples obtained during endoscopic retrograde cholangiopancreatography for malignant biliary strictures
Source: PLoS One. 2023 Aug 10;18(8):e0289537. doi: 10.1371/journal.pone.0289537 (PMC10414614; doi:10.1371/journal.pone.0289537)

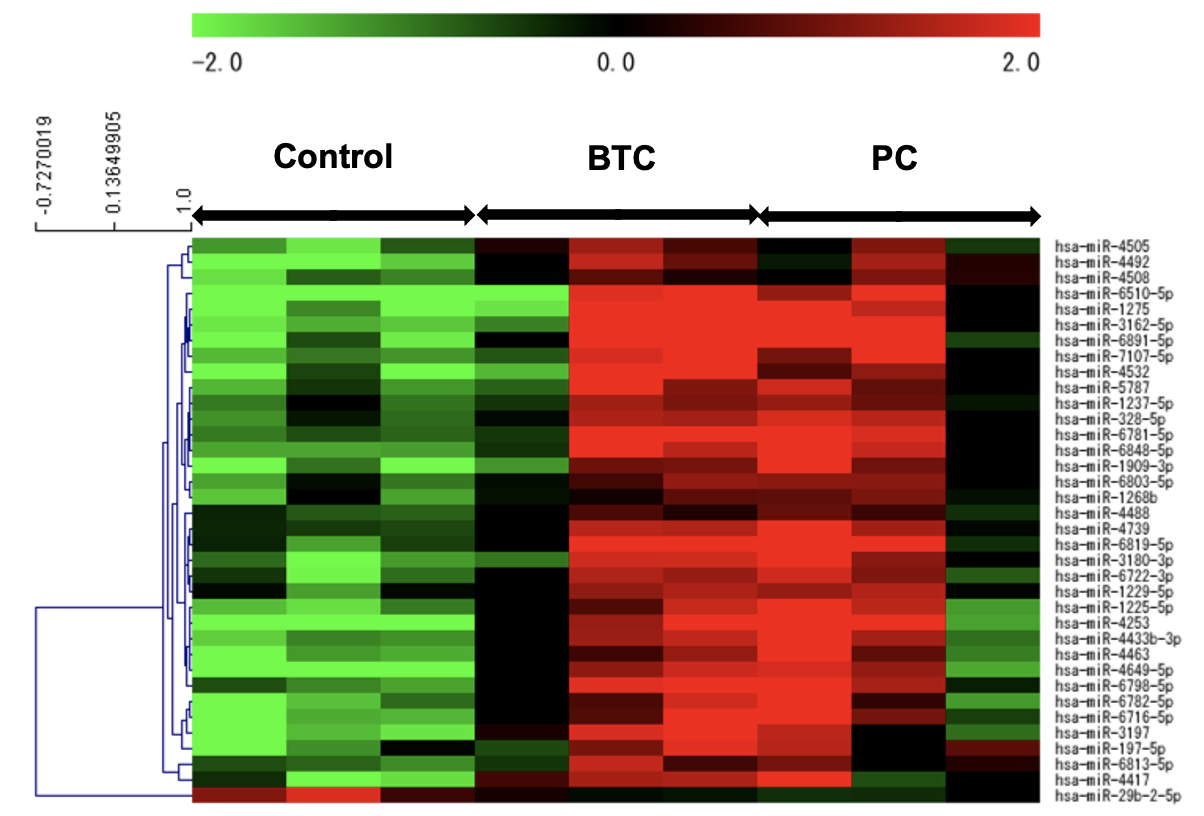

Supplement: S1 Fig — Heatmap representing miRNA expression in bile samples from 9 cases (3 pancreatic cancers, 3 biliary tract cancers, 3 controls). Thirty-five of 2578 miRNAs were significantly up-regulated, and one miRNA was significantly downregulated in PC and BTC bile samples compared with the control (P<0.05). (TIF) [file pone.0289537.s002.tif]

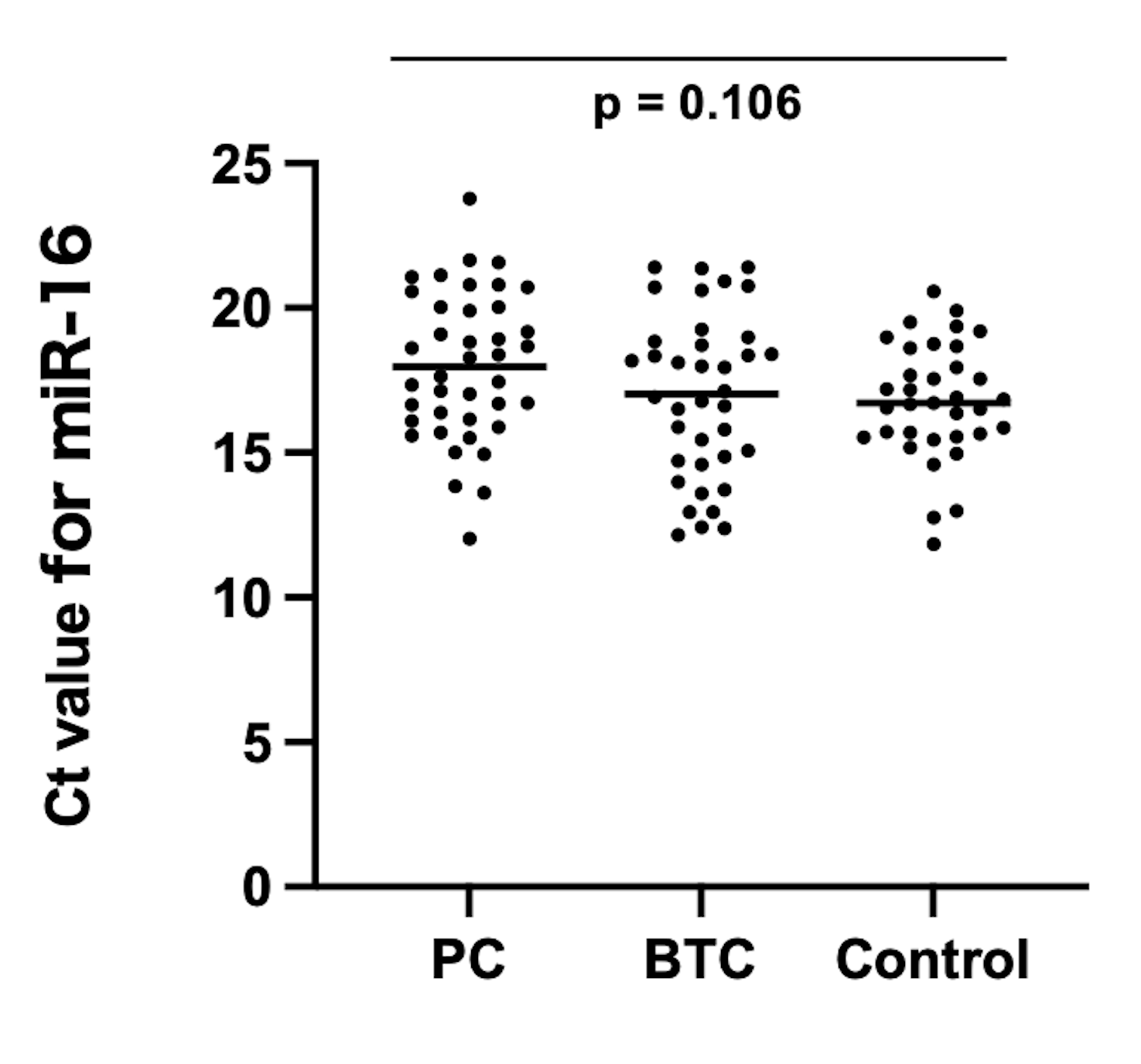

Supplement: S2 Fig — The Ct values for miR-16 were present in sufficient quantities and no significant differences (P > 0.05) in the 113 bile samples, thus validating miR-16 as a reliable endogenous housekeeping. (TIF) [file pone.0289537.s003.tif]

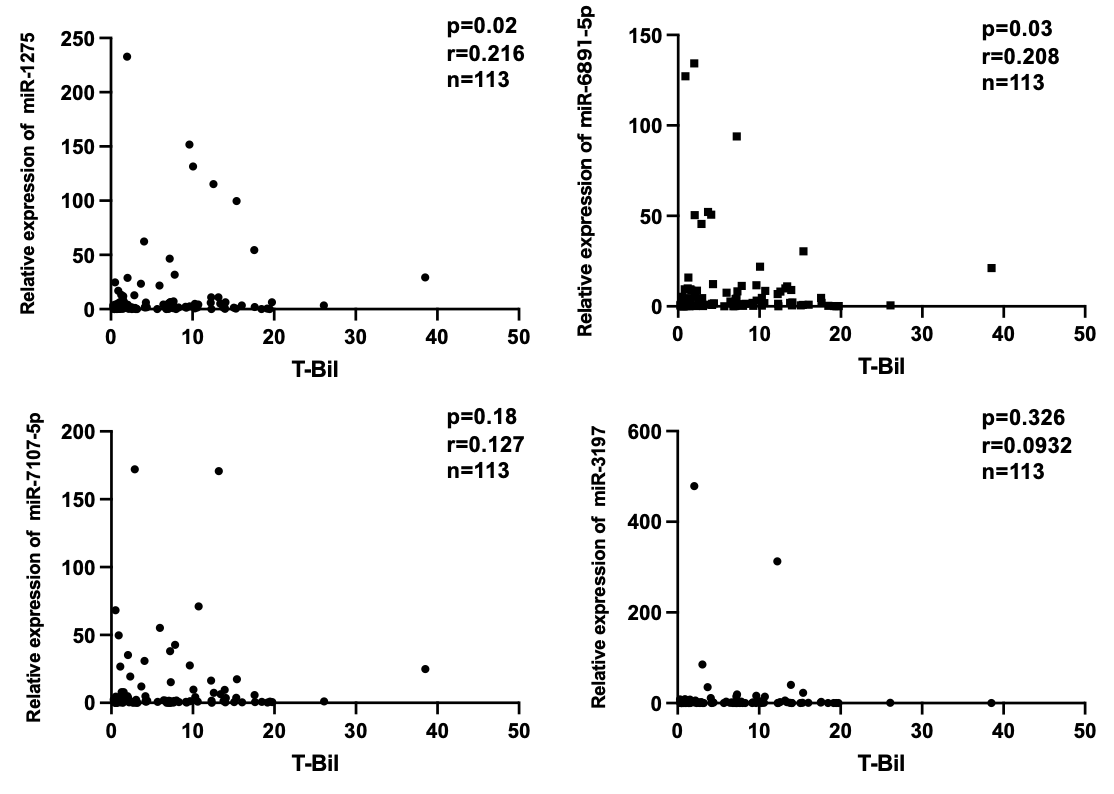

Supplement: S3 Fig — Correlation between the miRNA measurement and cholestasis analyzed by linear regression test (n = 113). No significant correlation was observed between the quantification of the miRNAs in bile and total bilirubin. (TIF) [file pone.0289537.s004.tif]

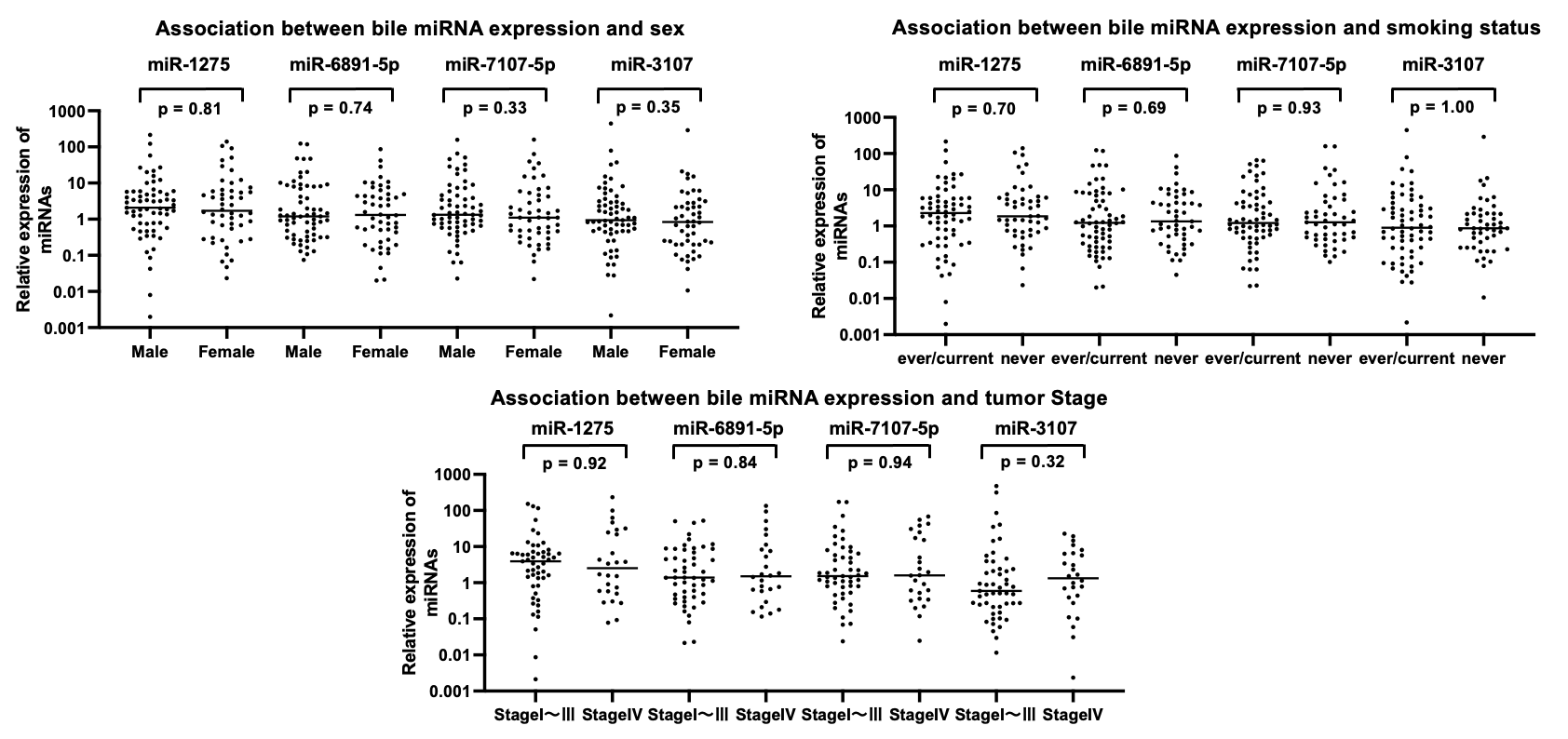

Supplement: S4 Fig — Differential expression of miRNA in bile is illustrated as a function of sex, smoking status (ever/current or never) and tumor stage. No significant differences in any miRNA expression levels were found in these clinical statuses. (TIF) [file pone.0289537.s005.tif]
