# Supplementary material for: Diagnostic utility of quantitative analysis of microRNA in bile samples obtained during endoscopic retrograde cholangiopancreatography for malignant biliary strictures
Source: PLoS One. 2023 Aug 10;18(8):e0289537. doi: 10.1371/journal.pone.0289537 (PMC10414614; doi:10.1371/journal.pone.0289537)
Supplement: S1 Table — (DOCX) [file pone.0289537.s006.docx]

|  | Average signal | |  |  |
| --- | --- | --- | --- | --- |
|  | control | PC+BTC | Fold-change | P-value |
| miR-3197 | 29.4 | 179.8 | 6.11 | 0.004 |
| miR-6891-5p | 4.7 | 222.9 | 47.5 | 0.008 |
| miR-3162-5p | 8.1 | 154.3 | 19.2 | 0.008 |
| miR-1275 | 6.2 | 119.4 | 19.4 | 0.009 |
| miR-4532 | 89.9 | 744.4 | 8.31 | 0.010 |
| miR-7107-5p | 65.3 | 398.9 | 6.13 | 0.013 |

PC: pancreatic cancer, BTC: Biliary tract cancer;
