# Supplementary material for: Diagnostic utility of quantitative analysis of microRNA in bile samples obtained during endoscopic retrograde cholangiopancreatography for malignant biliary strictures
Source: PLoS One. 2023 Aug 10;18(8):e0289537. doi: 10.1371/journal.pone.0289537 (PMC10414614; doi:10.1371/journal.pone.0289537)
Supplement: S2 Table — (DOCX) [file pone.0289537.s007.docx]

| hsa-miR- 1275  5 ́- GUGGGGGAGAGGCUGUC- 3 ́  hsa-miR-6891-5p  5 ́- UAAGGAGGGGGAUGAGGGG-3 ́  hsa-miR-7107-5p  5 ́- UCGGCCUGGGGAGGAGGAAGGG- 3 ́  has-miR-3197  5 ́- GGAGGCGCAGGCUCGGAAAGGCG- 3 ́ |
| --- |
