# Supplementary material for: Diagnostic utility of quantitative analysis of microRNA in bile samples obtained during endoscopic retrograde cholangiopancreatography for malignant biliary strictures
Source: PLoS One. 2023 Aug 10;18(8):e0289537. doi: 10.1371/journal.pone.0289537 (PMC10414614; doi:10.1371/journal.pone.0289537)
Supplement: Attachment — Submitted filename: Response to Reviewers.docx [file pone.0289537.s008.docx]

Thank you very much for providing important insights. In the following sections, you will find our responses to each of your points and suggestions. We are grateful for the time and energy you expended on our behalf.

Additional Editor Comments:

This paper is well written and the data are well presented. I think this paper quality can be improved if authors can provide more evidence such as bioinformatics analysis from TCGA.

Reviewer #1: 
I have some comments:
- Although significant, the differences between tumor patients and the controls is low talking about Ct. Which would be the threshold to consider that a blind bile sample analyzed has the miRNA high or low and therefore would be consider as malignant or benign?

A. Using the Youden index, the threshold for miR-1275 to differentiate pancreatic cancer from controls was 1.57 and the threshold for miR-6891-5p was 0.55. The threshold for miR-1275 to differentiate biliary tract cancer from controls was 3.28. We added the information at “Quantitative analysis of bile miRNAs in patients with PC, BTC and control.” in page 16- lines 243,246,248.

- Although in Material&Methods it is described (page 7, lines 96-98), I am not sure I understood it correctly. Patients included in the study were diagnosed with a malignancy in the samples collected during the ERCP. According to Fig 1 there are control samples with high levels of miRNAs (comparable to tumor patients). Have you follow these patients to know whether they were later diagnosed with a tumor? This would really improve the utility you would anticipate the diagnosis.

A. Yes, we followed these patients for at least one year. Not all of them had negative pathological results later, but all of them had a clinical course and multimodal imaging tests, including computed tomography, endosonography and/or magnetic resonance imaging, which confirmed the absence of new lesions suspicious for malignancy. Therefore, we believe that control samples with high levels of miRNAs are not malignant.

- Authors have used miR-16 as housekeeping for the qPCR analysis and all the conclusions are based on those results. Although they justify that other authors used miR-16 as housekeeping, the other studies were done in plasma or tissue samples. Authors should include a supplementary figure reporting the miR-16 value for the 113 samples analysed to demonstrate that it is a good housekeeping.

A. It was observed that the CT values for miR-16 were present in sufficient quantities and no significant differences (P > 0.05) in the 113 bile samples, thus validating miR-16 as a reliable endogenous housekeeping. We added the date in a flowchart (supplementary figure2) and information at “Results” in page 16- lines 234-236.

- I found contradictory that in page 25- lines 380-383 authors said that there is one 1 paper about miRNA in bile for PC diagnosis (the ref 14), and then in page 29- lines448-451 they said that there are several reports on qPCR analysis of miRNAs in bile. The difference is the type of tumor? I mean that in these “several reports” they do not have PC patients? Or what? Moreover, references for these statement in page 29 are missing.

A. I apologize for the confusing description. As you say, the difference is the type of tumor. Several reports listed in page 29- lines448-451 include one paper on PC and several papers on BTC. We added the reference lists in page 29- lines 462.

- I also find weird that contradictory results are found between this study and Lei et al in terms of miR-1275 levels in PC patients (REF 38). Of course as authors discussed I agree with the difference in the sample used (tissue vs bile), but normally there should not be these discrepancy. I think it would be important to analyzed some (n=10-15) pair samples (tissue-bile) in your hands.

A. Unfortunately, we do not have pair sample readily available, so we are unable to perform the actual validation. However, there are some studies with the contradictory results for the same type of miRNA depending on the type of sample used. Flammang et al. showed a downregulation of tissue-derived miR-192-5p and an upregulation of blood miR-192-5p in patients with pancreatic ductal adenocarcinoma compared to healthy controls. They state that the reason may involve not only benign and malignant factors, but also multifactorial involvement, such as the presence of inflammation.　Including this study, there are only two reports of quantitative analysis of miR-1275 in PC. Therefore, we believe that a certain trend will be seen as more studies are accumulated. We added the information at “Discussion.” in page 28- lines 440-449.

- To perform the miRNA expression analysis 130 ng of total RNA from each sample was used. Does this mean that 45 ng of each of the 3 samples were pooled?

A. No, we used 130ng of total RNA from each of the 9 samples to perform the miRNA expression analysis by microarray. We changed the statement in the text.

- Titles in Results section should be more informative than a simple “Patients” or “Quantitative PCR”.

A. We changed the titles in Result section “patients” to “Study population and characteristics” (page 13- lines 196) and “Quantitative PCR” to “Quantitative analysis of bile miRNAs in patients with PC, BTC and control” (page 16- lines 231-232).

Again, thank you for giving us the opportunity to strengthen our manuscript with your valuable comments and queries. We have worked hard to incorporate your feedback and hope that these revisions persuade you to accept our submission.
